# Salivary Cortisol Determination in ACTH Stimulation Test to Diagnose Adrenal Insufficiency in Patients with Liver Cirrhosis

**DOI:** 10.1155/2019/7251010

**Published:** 2019-06-20

**Authors:** Lara Albert, Joaquím Profitós, Jordi Sánchez-Delgado, Ismael Capel, José Miguel González-Clemente, David Subías, Albert Cano, Eugenio Berlanga, Anna Espinal, Marta Hurtado, Rocío Pareja, Mercedes Rigla, Blai Dalmau, Mercedes Vergara, Mireia Miquel, Meritxell Casas, Olga Giménez-Palop

**Affiliations:** ^1^Endocrinology Department, Parc Taulí University Hospital, Institut d'Investigacio i Innovació Parc Taulí I3PT, Autonomous University of Barcelona, Sabadell, Spain; ^2^Liver and Gastrointestinal Diseases Department, Parc Tauli University Hospital, Institut d'Investigacio i Innovació Parc Taulí I3PT, Autonomous University of Barcelona, Sabadell, Spain; ^3^Centro de Investigación Biomédica en Red de Enfermedades Hepáticas y Digestivas (CIBERehd), Instituto de Salud Carlos III, Spain; ^4^Clinical Laboratory Department, Institut d'Investigació i Innovació Parc Taulí I3PT, Parc Taulí University Hospital, Autonomous University of Barcelona, Sabadell, Spain; ^5^Servei d'Estadística Aplicada, Universitat Autònoma de Barcelona, Bellaterra, Spain

## Abstract

**Purpose:**

The prevalence of adrenal insufficiency (AI) in patients with decompensated liver cirrhosis is unknown. Because these patients have lower levels of cortisol-binding carrier proteins, their total serum cortisol (TSC) correlates poorly with free serum cortisol (FC). Salivary cortisol (SaC) correlates better with FC. We aimed to establish SaC thresholds for AI for the 250 *μ*g intravenous ACTH test and to estimate the prevalence of AI in noncritically ill cirrhotic patients.

**Methods:**

We included 39 patients with decompensated cirrhosis, 39 patients with known AI, and 45 healthy volunteers. After subjects fasted ≥8 hours, serum and saliva samples were collected for determinations of TSC and SaC at baseline 0'(T_0_) and at 30-minute intervals after intravenous administration of 250 *μ*g ACTH [30'(T_30_), 60'(T_60_), and 90'(T_90_)].

**Results:**

Based on the findings in healthy subjects and patients with known AI, we defined AI in cirrhotic patients as SaC-T_0_< 0.08 *μ*g/dL (2.2 nmol/L), SaC-T_60_ < 1.43 *μ*g/dl (39.5 nmol/L), or ΔSaC<1 *μ*g/dl (27.6 nmol/L). We compared AI determination in cirrhotic patients with the ACTH test using these SaC thresholds versus established TSC thresholds (TSC-T_0_< 9 *μ*g/dl [248 nmol/L], TSC-T_60_ < 18 *μ*g/dl [497 nmol/L], or ΔTSC<9 *μ*g/dl [248 nmol/L]). SaC correlated well with TSC. The prevalence of AI in cirrhotic patients was higher when determined by TSC (48.7%) than by SaC (30.8%); however, this difference did not reach statistical significance. AI was associated with sex, cirrhosis etiology, and Child-Pugh classification.

**Conclusions:**

Measuring SaC was more accurate than TSC in the ACTH stimulation test. Measuring TSC overestimated the prevalence of AI in noncritically ill cirrhotic patients.

## 1. Introduction

Adrenal insufficiency (AI) is common in patients with liver disease; AI is present in both patients with severe cirrhosis admitted to intensive care units and stable patients [1–3]. Relative AI in patients with cirrhosis is a sum of primary (lack of steroid precursors, such as cholesterol) and secondary (impairment of CRH-ACTH axis) AI. The lack of specific symptoms of acute and chronic AI makes the diagnosis difficult.

The insulin tolerance test is considered the gold standard for evaluating the hypothalamus-pituitary–adrenal axis. However, in clinical practice, the short ACTH stimulation test is more widely used because it is better tolerated and has fewer contraindications. Both tests are based on the analysis of serum cortisol, and the correlation between the two is well studied; most clinical guidelines support the use of the ACTH test for diagnosing AI [4, 5].

Serum cortisol is mostly bound to carrier proteins such as cortisol-binding globulin (CBG) and albumin [6]. Free cortisol (FC), the biologically active unbound fraction, represents about 5% to 10% of total serum cortisol (TSC) [7, 8]. Various conditions affect protein synthesis. For instance, cirrhosis, malnutrition, and critical illness reduce it, whereas oral contraceptives and pregnancy increase it. Thus, TSC does not accurately reflect FC, increasing the risk of misdiagnosis [9–11]. Rauschecker et al. [12] recently demonstrated that measuring FC in response to ACTH stimulation is a good alternative to TSC for diagnosing AI. However, FC analysis is time-consuming and expensive, hindering its use for routine laboratory testing. The FC fraction can be calculated using the Coolens' equation, but the results are unsatisfactory [13]. An easier, less expensive approach is to determine FC indirectly by measuring salivary cortisol (SaC), a surrogate of plasma FC [14].

Late-night (23:00–24:00 h) SaC is widely used to detect hypercortisolism when Cushing's syndrome is suspected [15]. Various authors have proposed using SaC instead of TSC after ACTH stimulation tests [15–17], but limited data are available to validate this approach.

We aimed to determine the reference values for SaC after stimulation with 250 *μ*g intravenous ACTH, to determine the diagnostic accuracy of these values for AI in noncritical patients with cirrhosis, and to estimate the prevalence of AI in this population.

## 2. Materials and Methods

Subjects were enrolled from April 2013 through October 2015. We included (a) 45 healthy adults recruited from hospital staff (17 men; mean age, 30 years; range, 22–49 years); none required medication within 1 month of testing, and all had normal liver, renal, and thyroid function; (b) 41 endocrinology patients with known AI diagnosed by insulin tolerance test or short 250*μ*g ACTH test (12 men; mean age, 57 years; range, 24–86 years; 13 primary AI, 26 secondary AI); and (c) 39 noncritical cirrhotic patients hospitalized for cirrhosis-related complications (34 men; mean age, 58 years; range, 39–90 years). Cirrhosis was diagnosed through histological or clinical, biological, and ultrasonographic findings.  Table 1 reports cirrhotic patients' demographic and clinical data. Reasons for hospitalization were ascites, gastrointestinal bleeding, infection without systemic inflammatory response syndrome, alcoholic hepatitis, acute kidney injury, and others.

Exclusion criteria were age <18 years; pregnancy; use of glucocorticoids (except in AI patients) or oral contraceptives < 6 months before inclusion; severe acute illness; mean arterial pressure < 60 mmHg; blood in the mouth; administration of albumin, fresh frozen plasma, or terlipressin before inclusion; or absence of consent.

At inclusion, patients were examined, with special attention to the presence of blood in the mouth. Patients were told not to brush their teeth, smoke, or drink anything but water during the 60 min before sampling. Patients on chronic corticosteroid replacement therapy received their last dose at 9:00 a.m. the day before testing.

The first sample was extracted between 8:30 a.m. and 9:30 a.m. after at least 8h fasting. To avoid stress-induced bias, baseline (T_0_) samples were obtained 30 minutes after catheterization of a superficial vein. Blood samples were drawn from the catheter. Saliva samples were collected after patients chewed a cotton swab specially designed for cortisol determination from saliva (Salivette®, Sarstedt AG&Co; Nümbrecht, Germany) for 1 to 3 minutes. After 250 *μ*g of synthetic ACTH (Synacthen®, Alfasigma; Milan, Italy) was administered intravenously, blood and saliva samples were collected at minutes 30 (T_30_), 60 (T_60_), and 90 (T_90_). All samples were processed immediately.

SaC and TSC levels were determined by electrochemiluminescence assay (Roche Diagnostics GmbH; Mannheim, Germany) [lower limit of detection, 0.018 *μ*g/dL (0.50 nmol/L); coefficient of variation, 4.1%–4.9% at high levels and 7.5%–11.5% at low levels]. Values of SaC or TSC <0.018 *μ*g/dL (0.50 nmol/L) were excluded from the analyses.

For the diagnosis of AI, we used the following established cutoffs: TSC T_0_< 9 *μ*g/dl (248 nmol/L), TSC T_60_ < 18 *μ*g/dl (497 nmol/L), or ΔTSC (increase between T_0_ and T_60_) <9 *μ*g/dl (248 nmol/L) [4, 18–20]. Salivary cortisol cutoffs were defined as the minimum SaC concentration observed in healthy subjects at T_0_ and T_60_ and the minimum ΔSaC value. We analyzed the correlation between TSC levels and SaC levels. We used the SaC cutoffs and TSC cutoffs to assess the prevalence of AI in the cirrhotic group and compared the results obtained with the two methods.

We did a descriptive analysis of patients' clinical characteristics. We used descriptive statistics to summarize the values of TSC and SaC at each timepoint and the differences between their values at baseline and 60 minutes (ΔTSC and ΔSaC). Using these statistics, we defined three criteria for the diagnosis of AI. The likelihood-ratio test was used to check the goodness of fit.

We used SAS 9.3 (SAS System, Cary, NC, USA, 2013) for all analyses.

## 3. Results

### 3.1. Healthy and AI Subjects

**Figure 1:**
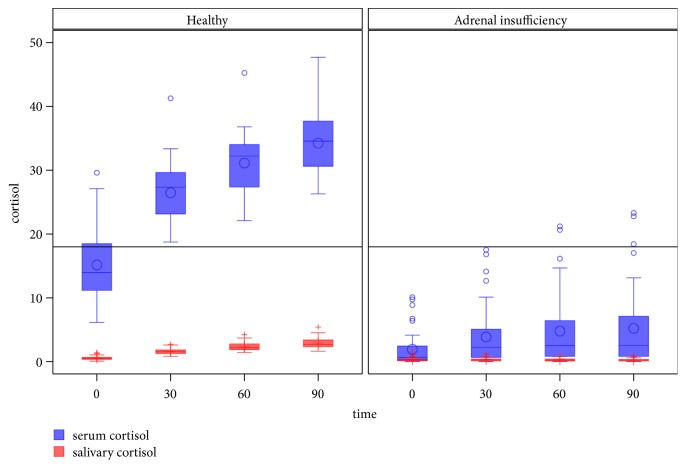
Serum and salivary cortisol (*μ*g/dL) in the 250 *μ*g ACTH test. To convert cortisol micrograms per deciliter to nanomoles per liter, multiply by 27.6.

All healthy controls had TSC ≥18 *μ*g/dL (497 nmol/L) at T_60_; two AI subjects surpassed this cutoff and were excluded from the analyses.  Figure 1 shows the distribution of TSC and SaC after ACTH stimulation. As expected, all TSC determinations were lower in AI subjects than in healthy subjects (Table 2). Mean ΔTSC was 15.97±4.80 *μ*g/dL (441±132 nmol/L) in healthy subjects and 2.84±3.14 *μ*g/dL (78±87 nmol/L) in AI patients (Table 3).

In healthy subjects, mean SaC at T_0_ was 0.56 ± 0.31*μ*g/dL (15±9 nmol/L); the lowest value was 0.08 *μ*g/dL (2.2 nmol/L) (Table 4). The area under the receiver operating characteristic curve for SaC-T_0_ was 0.8045. After ACTH stimulation, SaC progressively increased in nearly all healthy subjects; the lower limit of SaC at T_60_ was 1.43 *μ*g/dL (39.5 nmol/L) (Table 4). The cutoff SaC at T_60_ > 1.43 *μ*g/dL (39.5 nmol/L) classified all AI patients correctly.

In healthy subjects, mean ΔSaC was 1.79±0.59 *μ*g/dL (49.4±16.3 nmol/L); the lowest value was 1 *μ*g/dL (27.6 nmol/L) (Table 5). We defined these concentrations (SaC-T_0_< 0.08 *μ*g/dL [2.2 nmol/L], SaC-T_60_< 1.43 *μ*g/dL [39.5 nmol/L], ΔSaC<1 *μ*g/dL [27.6 nmol/L]) as cutoff values for the diagnosis of AI.

In AI patients, mean SaC at T_0_ was 0.33±0.30 *μ*g/dL (9.1±8.3 nmol/L); the highest value was 1.53*μ*g/dL (42.2 nmol/L) (Table 4). After ACTH stimulation, SaC in AI patients mainly remained constant over time (Table 4). The highest concentration of SaC at T_60_ in AI patients was 0.90*μ*g/dL (24.8 nmol/L); therefore, the SaC-T_60_ cutoff classified all AI patients correctly. Mean ΔSaC in AI patients was -0.004±0.18 *μ*g/dL (-0.11±5.0 nmol/L); all had ΔSaC values lower than 1 *μ*g/dL (27.6 nmol/L) (Table 5).

Table 6 reports the correlations between SaC and TSC concentrations at different timepoints during the ACTH test. In AI patients, SaC and TSC correlated except at baseline, and the strength of the correlations increased over time. By contrast, in healthy patients, SaC and TSC correlated strongly at all timepoints.

### 3.2. Cirrhotic Subjects

Mean values of TSC and SaC at the different timepoints in the ACTH test in cirrhotic patients are reported in Tables 2 and 4, respectively; mean values of ΔTSC and ΔSaC are reported in Tables 3 and 5, respectively. Based on the results for healthy subjects and AI patients, we selected the following cutoffs for the diagnosis of AI in cirrhotic patients SaC-T_0_< 0.08 *μ*g/dL (2.2 nmol/L) or SaC-T_60_< 1.43 *μ*g/dl (39.5 nmol/L) or ΔSaC<1 *μ*g/dl (27.6 nmol/L).

Table 7 reports the numbers of cirrhotic patients that met each criterion for the diagnosis of AI with each method. Comparing the results of using the SaC thresholds versus the established TSC thresholds to diagnose AI in cirrhotic patients, we found 19 patients met at least one TSC criterion of AI and 12 patients met at least one SaC criterion; however, this difference in frequency did not reach statistical significance. The criteria for AI according to both of the two methods were met by 11 (28.2%) patients; 8 (20.5%) met only the TSC criteria, and 1 (2.6%) met only the SaC criteria.

Based on these results, we classified cirrhotic patients into three groups: No AI (n=19), AI based on TSC and SaC criteria (n=12), and AI based on TSC but not SaC criteria (n=8). Comparing the characteristics of the patients in these groups, we found that the No-AI group had a higher proportion of women (X^2^=17.14; p<0.001), more patients with non-alcohol-related cirrhosis (although only 2 patients had non-alcohol-related cirrhosis), and a greater-than-expected proportion of Child A and Child B patients (X^2^=20.10; p=0.0005); both AI groups had a greater-than-expected proportion of patients with alcohol-related cirrhosis (X^2^=25.29; p=0.0014). We found no significant differences between the three groups in age at diagnosis of cirrhosis, current MELD, blood albumin, prealbumin, HDL or LDL cholesterol, creatinine, triglycerides, AST, ALT, bilirubin, prothrombin time, INR, or reasons for admission.Cirrhotic subjects with AI diagnosed according to SaC levels were treated with glucocorticoid replacement therapy.

## 4. Discussion

To determine whether SaC can be used for diagnosing AI in noncritical cirrhotic patients, we established reference values for SaC at T_0_ and T_60_ and for ΔSaC (T_0_-T_60_) based on SaC and TSC findings in normal subjects and patients with known AI. We found that SaC can be very useful for diagnosis of AI in cirrhotic patients.

Determining SaC is a quick, easy, noninvasive technique used since the early 1980s, when SaC was discovered to be an excellent indicator of plasma FC concentration [21, 22]. Various authors have since studied TSC and SaC in different circumstances in which cortisol-binding globulin is altered (oral contraception, pregnancy, and cirrhosis) [10, 23]. In 2009, Deutschbein et al. [23] compared basal SaC and basal TSC to the insulin tolerance test in 77 patients with hypothalamic-pituitary disease, concluding that both approaches enabled a highly specific diagnosis, obviating insulin tolerance testing in about one-fourth of cases. In 2012, they found basal SaC<0.11 *μ*g/dL (3.0 nmol/L) had 97% specificity and 40% sensitivity for AI, enabling correct classification in 26% [24]. By contrast, Ceccato et al. [25] concluded that unstimulated SaC<0.09 *μ*g/dL (2.5 nmol/L) distinguished AI patients from healthy subjects with 97.1% sensitivity and 93.3% specificity. Recently, Langelaan et al. [26] recommended a new diagnostic algorithm to diagnose AI, with early morning SaC>0.21 *μ*g/dL (5.8 nmol/L) ruling out AI and <0.04 *μ*g/dL (1.1 nmol/L) diagnosing AI; these cutoffs enabled 34% of patients to be diagnosed without ACTH stimulation. In our study, SaC-T_0_< 0.08 *μ*g/dL (2.2 nmol/L) (the lowest SaC-T_0_ value in normal subjects) diagnosed 7.7% of patients with AI; the differences in cutoffs might be due to differences in laboratory methods for determining SaC.

In 1988, Laudat et al. [27] found no overlap in SaC-T_60_ after 250 *μ*g ACTH between 58 healthy volunteers and 21 subjects with AI; discrepancies between SaC and TSC in 8 patients with AI were attributed to thyroid hormones and psychotropic agents. Various studies have since examined correlations between TSC and SaC during 1 *μ*g or 250 *μ*g ACTH tests [16, 28–30]. Correlations between TSC and SaC are good; the issue is choosing the optimal SaC cutoff for AI. Methodological differences among studies make comparisons difficult. Some studies included healthy volunteers and patients with known AI, whereas others included subjects with suspected AI. Moreover, some used 1 *μ*g ACTH, whereas others used 250 *μ*g. Finally, different studies used different methods to measure SaC and TSC, and the normal response to ACTH tests is assay-specific [31]. Thus, despite the available data, clinicians face uncertainty in choosing cutoffs.

We compared SaC and TSC before and after administering 250 *μ*g ACTH in healthy subjects and patients with known AI to calculate reference values for basal SaC, peak SaC at T_60_, and ΔSaC (T_0_-T_60_). We used these references to determine the prevalence of AI in a cohort of decompensated cirrhotic patients and compared the results with those found using established TSC cutoffs. Whereas the established TSC cutoffs classified 48.7% of the cirrhotic patients as AI, our SaC cutoffs classified only 30.8% as AI. The difference was not statistically significant, probably due to the low number of cirrhotic patients, but it shows a clear tendency. At least one SaC criterion and one TSC criterion for AI were met in 11 (28.2%); 8 (20.5%) had AI according to TSC but not according to SaC, and 1 patient had AI according to SaC but not TSC. The 30.8% SaC-determined prevalence in our noncritical cirrhotic patients is higher than the 9.1% found by Galbois et al. [32]; it is also higher than the 19% prevalence calculated by Fede et al. [33] by measuring FC after a 1 *μ*g ACTH test. As in other studies, we found that TSC overestimated the prevalence of AI (48.7% in our study, 33% in Galbois et al. [32], and 34% in Fede et al. [33]). The higher prevalence of AI in our study might be due to greater liver disease severity (85% Child B or C); we found that severity was associated with a higher risk of AI. We also found that sex and cirrhosis etiology were associated with AI frequency.

Several studies identified ascites, low HDL-cholesterol, and liver disease severity as risk factors for AI [32, 34–36]; other reported risk factors include low cortisol-binding globulin [33], higher MELD score [37], and lower serum albumin [32]. Galbois et al. [32] reported that hypoalbuminemia was the main reason for discrepancies between TSC and SaC assessments of AI, suggesting that a lower threshold of 25 g/L could be used to identify patients who could benefit from a SaC assessment. When we analyzed the subgroup of patients with albumin<25 g/L, we found no differences in the prevalence of AI measured by SaC or TSC, probably because few (8/39) patients had albumin<25 g/L.

Our study has several limitations. We calculated disease-specific thresholds of SaC measured by electrochemiluminescence immunoassay, the method routinely used in our hospital. Liquid chromatography tandem-mass spectrometry is more specific but requires expensive equipment [38]. Despite the risk of overestimating SaC, potential cross-reactivity with other steroids, and different results obtained with different types of analyzers [39, 40], we consider the use of electrochemiluminescence immunoassay justified because it is the method most commonly used in clinical practice. Moreover, we did not measure FC, because doing so is complex, expensive, and uncommon in clinical practice; likewise, we did not measure cortisol-binding globulin, so we could not calculate FC with Coolens' equation.

In diagnostic accuracy studies, how eligible subjects are identified and recruited is important. We included only hemodynamically stable cirrhotic patients, so our findings cannot be extrapolated to cirrhotic patients with sepsis or septic shock. On the other hand, we determined cutoffs from our findings in patients with known AI and healthy volunteers with low probability of AI, adding strength to our results. Other studies derived cutoffs from findings in patients with suspected AI, and that design could influence the spectrum of disease in included patients [41]. Moreover, test sensitivity is usually higher in studies with patients with more advanced stages of the target condition [42].

Measuring SaC has some methodological limitations. SaC's concentration is 30-fold lower than TSC's. Additionally, SaC levels are affected by salivary 11*β*-hydroxysteroid dehydrogenase type 2. In 2010, Perogamvros et al. [43] suggested using salivary cortisone better reflects FC after adrenal stimulation. Cornes et al. [40] report that concentrations of salivary cortisone are three times higher than those of SaC and have a closer linear correlation with serum FC. Debono et al. [44] also suggested that salivary cortisone may be the preferred analyte for noninvasive measurement of FC.

## 5. Conclusions

We establish method-specific reference cutoffs of SaC and ΔSaC to determine AI during the 250 *μ*g ACTH stimulation test. SaC is more accurate than TSC for assessing adrenal function with the 250 *μ*g ACTH stimulation test in noncritical cirrhotic patients. The prevalence AI in our cirrhotic group was higher than in other studies. However, it should be taken into account that we did not measure CBG levels. Further studies with more cases are necessary to establish SaC reference values to correctly classify patients with AI and avoid unnecessary cortisol replacement treatment.

## Figures and Tables

**Table 1:** Clinical characteristics of cirrhotic patients.

Variable	Data
Age, years*∗*	58.5 ± 11.7
Age at diagnosis of cirrhosis, years*∗*	53.8 ± 10.8
Male sex, n (%)	34 (87.2)
Cirrhosis etiology, n (%)	
Alcohol	30 (76.9)
Alcohol and HCV	5 (12.8)
Alcohol and HBV	2 (5.1)
Primary biliary cirrhosis	1 (2.6)
Autoimmune disorder	1 (2.6)
Type 2 diabetes, n (%)	9 (23)
Ascites/edema, n (%)	35 (89.7)
Encephalopathy, n (%)	9 (23.1)
Hypertension, n (%)	12 (30.8)
Child-Pugh score*∗*	9 ± 2
Class A/B/C, n (%)	6 (15.4)/18 (46.2)/15 (38.5)
MELD score*∗*	17 ± 6
Albumin, g/L*∗*	30.4 ± 6.4
Prealbumin, g/L*∗*	6.0 ± 2.4
Cholesterol, mg/dL^*δ*^	109 (83-134)
HDL cholesterol, mg/dL^*δ*^	28 (13-39)
LDL cholesterol, mg/dL^*δ*^	68 (50-78)
Triglycerides, mg/dL^*δ*^	72 (56-105)
AST, IU/L^*δ*^	47 (34-61)
ALT, IU/L^*δ*^	21 (15-31)
GGT, IU/L^*δ*^	108 (39-225)
Bilirubin, mg/dL^*δ*^	2.6 (1.6-5.8)
Prothrombin time*∗*	1.68 ± 0.91
INR*∗*	1.80 ± 0.98

*∗*Mean ± SD; ^*δ*^median (Q1-Q3).

**Table 2:** Serum cortisol concentrations in healthy volunteers, adrenal insufficient patients, and cirrhotic patients in the 250 *μ*g ACTH test.

		*Serum cortisol* (*μ*g/dL)						
		Mean	Median	SD	Min	Q1	Q3	Max
Group								
*Healthy*	T_0_	15.15	13.95	5.66	6.14	11.18	18.48	29.61
	T_30_	26.45	27.34	4.64	18.74	23.15	29.63	41.26
	T_60_	31.12	32.23	4.60	22.09	27.38	34.01	45.24
	T_90_	34.22	34.55	4.72	26.29	30.62	37.68	47.68

*Adrenal insufficiency*	T_0_	1.94	0.67	2.78	0.03	0.21	2.45	10.10
	T_30_	3.87	2.23	4.61	0.03	0.68	5.07	17.53
	T_60_	4.78	2.51	5.52	0.03	0.84	6.42	21.20
	T_90_	5.22	2.54	6.14	0.03	0.84	7.09	23.30

*Cirrhosis*	T_0_	12.75	12.27	5.91	2.05	8.20	17.80	24.85
	T_30_	21.52	22.35	6.79	11.02	16.35	26.33	40.20
	T_60_	26.23	26.73	7.93	13.83	18.59	32.96	42.76
	T_90_	29.94	29.89	9.01	15.28	21.31	38.17	45.67

T_0_, baseline; T_30_, 30 min after administration; T_60_, 60 min after administration; T_90_, 90 min after administration. To convert cortisol micrograms per deciliter to nanomoles per liter, multiply by 27.6.

**Table 3:** Change in serum cortisol between baseline and 60 minutes in healthy volunteers, adrenal insufficient patients, and cirrhotic patients in the 250 *μ*g ACTH test.

	*Delta serum cortisol* (*μ*g/dL)						
	Mean	Median	Std	Min	Q1	Q3	Max
Group							
*Healthy*	15.97	15.63	4.80	7.31	12.30	19.63	25.34
*Adrenal insufficiency*	2.83	1.73	3.14	-0.04	0.19	5.17	12.33
*Cirrhosis*	13.48	13.06	5.36	4.23	8.70	16.51	25.56

To convert cortisol micrograms per deciliter to nanomoles per liter, multiply by 27.6.

**Table 4:** Salivary cortisol concentrations in healthy volunteers, adrenal insufficient patients, and cirrhotic patients in the 250 *μ*g ACTH test.

		*Salivary cortisol* (*μ*g/dL)						
		Mean	Median	Std	Min	Q1	Q3	Max
Group								
*Healthy*	T_0_	0.56	0.52	0.31	*0.08*	0.37	0.68	1.37
	T_30_	1.58	1.56	0.45	0.83	1.28	1.83	2.72
	T_60_	2.35	2.19	0.63	1.43	1.89	2.78	4.24
	T_90_	2.92	2.71	0.82	*1.63*	2.35	3.40	5.42

*Adrenal insufficiency*	T_0_	0.33	0.26	0.30	0.05	0.13	0.36	1.53
	T_30_	0.32	0.29	0.24	0.05	0.13	0.41	1.17
	T_60_	0.32	0.27	0.24	0.05	0.12	0.42	0.90
	T_90_	0.37	0.28	0.50	0.05	0.11	0.37	3.13

*Cirrhosis*	T_0_	0.72	0.69	0.42	0.14	0.35	0.99	1.88
	T_30_	1.47	1.14	0.96	0.37	0.80	1.73	5.19
	T_60_	2.25	1.97	1.20	0.58	1.47	2.90	5.37
	T_90_	3.12	2.67	1.93	0.70	1.97	3.87	8.58

To convert cortisol micrograms per deciliter to nanomoles per liter, multiply by 27.6.

**Table 5:** Change in salivary cortisol between baseline and 60 minutes in healthy volunteers, adrenal insufficient patients, and cirrhotic patients in the 250 *μ*g ACTH test.

	*Delta salivary cortisol* (*μ*g/dL)						
	Mean	Median	Std	Min	Q1	Q3	Max
Group							
*Healthy*	1.79	1.67	0.59	*0.99*	1.38	2.03	3.61
*Adrenal insufficiency*	-0.00	0.00	0.18	-0.72	-0.02	0.06	0.35
*Cirrhosis*	1.53	1.33	0.99	0.08	0.80	1.98	4.65

To convert cortisol micrograms per deciliter to nanomoles per liter, multiply by 27.6.

**Table 6:** Pearson correlation (r) between serum and salivary cortisol concentrations in healthy volunteers and adrenal insufficient patients at different timepoints during the ACTH test.

HV SaC						AI SaC			
		T_0_	T_30_	T_60_	T_90_	T_0_	T_30_	T_60_	T_90_
HV TSC	T_0_	0.83^a^	-	-	-	-	-	-	-
	T_30_	-	0.67^a^	-	-	-	-	-	-
	T_60_	-	-	0.54^b^	-	-	-	-	-
	T_90_	-	-	-	0.62^a^	-	-	-	-

AI TSC	T_0_	-	-	-	-	0.14^c^	-	-	-
	T_30_	-	-	-	-	-	0.49^b^	-	-
	T_60_	-	-	-	-	-	-	0.71^a^	-
	T_90_	-	-	-	-	-	-	-	0.79^a^

HV, healthy volunteers; AI, adrenal insufficiency; TSC, total serum cortisol; SaC, salivary cortisol; T_0_, baseline; T_30_, 30 min after administration; T_60_, 60 min after administration; T_90_, 90 min after administration.

^a^p <0.0001, ^b^p <0.01, and ^c^p >0.05.

**Table 7:** Number of cirrhotic patients meeting at least one criterion for the diagnosis of AI based on serum cortisol and salivary cortisol thresholds during 250 *μ*g ACTH test (N=39).

Criterion	AI based on TSC	AI based on SaC
AI defined from T_0_, n	13^a^	0^d^
AI defined from T_60_, n	9^b^	9^e^
AI defined from the difference between 0 and 60 min, n	10^c^	12^f^
Total number of patients reaching at least one of the three criteria of adrenal insufficiency, n (%)	19 (48.7%)	12 (30.8%)

AI, adrenal insufficiency; TSC, total serum cortisol; SaC, salivary cortisol; T_0_, baseline; T_60_, 60 min after administration. To convert cortisol micrograms per deciliter to nanomoles per liter, multiply by 27.6.

^a^T_0_ TSC <9 *μ*g/dL, ^b^T_60_ TSC <18 *μ*g/dL, ^c^ΔTSC<9 *μ*g/dL.

^d^T_0_ SaC<0.08 *μ*g/dL, ^e^T_60_ SaC<1.43 *μ*g/dL, ^f^ΔSaC<1 *μ*g/dL.

## Data Availability

The data used to support the findings of this study are available from the corresponding author upon request.
